# HPV8-E6 Interferes with Syntenin-2 Expression through Deregulation of Differentiation, Methylation and Phosphatidylinositide-Kinase Dependent Mechanisms

**DOI:** 10.3389/fmicb.2017.01724

**Published:** 2017-09-08

**Authors:** Benjamin Marx, Daliborka Miller-Lazic, John Doorbar, Slawomir Majewski, Kay Hofmann, Martin Hufbauer, Baki Akgül

**Affiliations:** ^1^Institute of Virology, University of Cologne Cologne, Germany; ^2^Department of Pathology, University of Cambridge Cambridge, United Kingdom; ^3^Department of Dermatology and Venereology, Medical University of Warsaw Warsaw, Poland; ^4^Institute for Genetics, University of Cologne Cologne, Germany

**Keywords:** human papillomavirus (HPV), E6 oncoprotein, Syntenin-2, methylation, differentiation, phosphatidylinositol-4, 5-bisphosphate

## Abstract

The E6 oncoproteins of high-risk human papillomaviruses (HPV) of genus alpha contain a short peptide sequence at the carboxy-terminus, the PDZ binding domain, with which they interact with the corresponding PDZ domain of cellular proteins. Interestingly, E6 proteins from papillomaviruses of genus beta (betaPV) do not encode a comparable PDZ binding domain. Irrespective of this fact, we previously showed that the E6 protein of HPV8 (betaPV type) could circumvent this deficit by targeting the PDZ protein Syntenin-2 through transcriptional repression (Lazic et al., 2012). Despite its high binding affinity to phosphatidylinositol-4,5-bisphosphate (PI(4,5)P_2_), very little is known about Syntenin-2. This study aimed to extend the knowledge on Syntenin-2 and how its expression is controlled. We now identified that Syntenin-2 is expressed at high levels in differentiating and in lower amounts in keratinocytes cultured in serum-free media containing low calcium concentration. HPV8-E6 led to a further reduction of Syntenin-2 expression only in cells cultured in low calcium. In the skin of patients suffering from Epidermodysplasia verruciformis, who are predisposed to betaPV infection, Syntenin-2 was expressed in differentiating keratinocytes of non-lesional skin, but was absent in virus positive squamous tumors. Using 5-Aza-2′-deoxycytidine, which causes DNA demethylation, Syntenin-2 transcription was profoundly activated and fully restored in the absence and presence of HPV8-E6, implicating that E6 mediated repression of Syntenin-2 transcription is due to promoter hypermethylation. Since Syntenin-2 binds to PI(4,5)P_2_, we further tested whether the PI(4,5)P_2_ metabolic pathway might govern Syntenin-2 expression. PI(4,5)P_2_ is generated by the activity of phosphatidylinositol-4-phosphate-5-kinase type I (PIP5KI) or phosphatidylinositol-5-phosphate-4-kinase type II (PIP4KII) isoforms α, β and γ. Phosphatidylinositide kinases have recently been identified as regulators of gene transcription. Surprisingly, transfection of siRNAs directed against PIP5KI and PIP4KII resulted in higher Syntenin-2 expression with the highest effect mediated by siPIP5KIα. HPV8-E6 was able to counteract siPIP4KIIα, siPIP4KIIβ and siPIP5KIγ mediated Syntenin-2 re-expression but not siPIP5KIα. Finally, we identified Syntenin-2 as a key factor regulating PIP5KIα expression. Collectively, our data demonstrates that Syntenin-2 is regulated through multiple mechanisms and that downregulation of Syntenin-2 expression may contribute to E6 mediated dedifferentiation of infected skin cells.

## Introduction

Papillomaviruses are a family of non-enveloped DNA tumor viruses that infect the skin or mucosa of their vertebrate hosts (Bravo et al., 2010). Human papillomaviruses (HPV) are associated with the development of epithelial cancers and high-risk HPV of genus alpha (alphaPV) have been proven to be a necessary causative factor for cervical and oropharyngeal cancer (Hübbers and Akgül, 2015). HPV types of genus beta (betaPV) have been implicated in the development of cutaneous squamous cell carcinomas (SCC) and actinic keratosis. The association between betaPV and skin cancer was first reported in patients with Epidermodysplasia verruciformis (EV) (Majewski and Jablonska, 2006). Patients suffering from this condition have a specific susceptibility to infections with betaPV, which is characterized by virus-positive cutaneous lesions. These lesions mainly occur in sun-exposed areas and harbor a considerable risk of malignant transformation into SCC in affected individuals (Akgül et al., 2006; Borgogna et al., 2012; Howley and Pfister, 2015). Viral gene expression and replication proceed in a tightly controlled fashion regulated by keratinocyte differentiation, and lead to the expression of several non-structural viral early (E) proteins. Expression of the viral E4 protein during a productive infection contributes to virus release by perturbing the cytokeratin network. However, it may also represent a marker for viral infection during betaPV-associated skin cancer progression in EV patients (Borgogna et al., 2012; Quint et al., 2015). The HPV E6 and E7 oncoproteins are necessary for malignant conversion (Wallace and Galloway, 2015). A main feature of E6 oncoproteins from high-risk alphaHPV, e.g., HPV16, is their PDZ binding motif [identified as a domain interacting with PSD-95, Dlg, and ZO-1 proteins (hence the name PDZ (Ganti et al., 2015))], through which they promote the proteasomal degradation of a variety of cellular substrates harboring PDZ domains. Such PDZ proteins play a major role in maintenance of cell polarity, growth and differentiation as well as scaffolding proteins in multi-protein signaling complexes (Pim et al., 2012). Their degradation by the HPV E6 protein may cause loss of cell polarity and morphological conversion accompanied by an epithelial-mesenchymal transition, leading to transformation and carcinogenesis (Yugawa and Kiyono, 2009). The ability of E6 to bind to PDZ proteins correlates with its oncogenic potential (James and Roberts, 2016). In addition, the interactions between E6 and PDZ proteins also results in a significant stabilization of the E6 protein, which may ensure that sufficient E6 levels are maintained (Nicolaides et al., 2011). As previously mentioned, E6 of betaPV, e.g., HPV8, do not encode for a PDZ binding motif. We recently found, that the PDZ protein Syntenin-2, but not Syntenin-1, is transcriptionally downregulated in primary human keratinocytes by HPV8-E6. These results identified Syntenin-2 as the first PDZ protein being controlled by a betaHPV at mRNA level (Lazic et al., 2012). The PDZ protein family of Syntenins comprises Syntenin-1 and Syntenin-2. Syntenin-1 was originally identified as a protein that binds directly to the cytoplasmic domain of the syndecan family of heparan sulfate proteoglycans (Grootjans et al., 1997). These are implicated in cell adhesion and several growth factor signaling pathways (Zimmermann and David, 1999; Couchman, 2003; Egea-Jimenez et al., 2016; Fares et al., 2016). Although Syntenin-1 and -2 show different preferences, as far as protein binding is concerned, both proteins share the ability to interact with phosphatidylinositol-4, 5-bisphosphate (PI(4,5)P_2_) (Mortier et al., 2005). However, the precise mechanism by which Syntenin-2 is physiologically regulated and how transcription is modulated by HPV8-E6 remained elusive.

This study aimed to extend the understanding of transcriptional regulation controlling Syntenin-2 expression and how E6 interferes with these mechanisms. Our results suggest an interaction of E6 with Syntenin-2 expression through interference of cell differentiation, promoter-methylation and PI(4,5)P_2_ generating kinases.

## Materials and Methods

### Syntenin Sequence Analysis

For generation of the Syntenin evolutionary trees, representative Syntenin sequences were obtained from The UniProt Consortium (2017), aligned by the L-Ins-I algorithm of the MAFFT package (Katoh et al., 2002), and subjected to tree calculation by the T-Rex server (Boc et al., 2012) using the RAxML method (Stamatakis, 2006). Uniprot accession numbers for sequences analysis: human Syntenin-1 (SDCB1_HUMAN), human Syntenin-2 (SDCB2_HUMAN), murine Syntenin-1 (SDCB1_MOUSE), murine Syntenin-2 (SDCB2_MOUSE), zebrafish Syntenin-1 (Q6PUR1_DANRE), zebrafish Syntenin-2 (Q803J6_DANRE), xenopus Syntenin-1 (Q66JA3_XENLA), xenopus Syntenin-2 (Q801P2_XENLA), anolis Syntenin-1 (G1KE37_ANOCA), anolis Syntenin-2 (H9GDS9_ANOCA), chicken Syntenin-1 (Q5ZHM8_CHICK), chicken Syntenin-2 (R4GGF6_CHICK), mosquito Syntenin (Q7PK99_ANOGA), tick Syntenin (B7PNL5_IXOSC), nematostella Syntenin (A7S2D9_NEMVE), sponge Syntenin (I1FJB9_AMPQE), capsaspora Syntenin (A0A0D2VN74_CAPO3), monosiga Syntenin (A9UPR7_MONBE).

### Retroviral Expression Vectors and Infection of Keratinocytes

We have previously described the generation of the pLXSN-based retroviral vectors coding for HPV8-E6 (Akgül et al., 2010). PLXSN-HPV8-E6 based mutants were generated by site-directed mutagenesis using specific primers. For retroviral infection, 3 × 10^5^ cells were seeded into 6 cm dishes and treated as described in Hufbauer et al. (2013). Selection of infected cells was started 2 days later using 250 μg/ml G418. Positive clones were pooled and expanded.

### Cell Lines and Treatments

RTS3b (Purdie et al., 1993) and PM1 keratinocytes (Proby et al., 2000) were maintained in RM+ media [consisting of a 3:1 ratio of DMEM:F12 with 10% fetal bovine serum (FBS), 1% glutamine, 0.4 μg hydrocortisone, 10^-10^M cholera toxin, 5 μg/ml transferrin, 2 × 10^-11^M liothyronine, 5 μg/ml insulin, 10 ng/ml epidermal growth factor, 1× Penicillin/Streptomycin mixture] (Akgül et al., 2003). HaCaT cells (Boukamp et al., 1988) were grown in DMEM with 10% FBS. N/TERT cells (Dickson et al., 2000) were either cultivated in KGM-Gold (which contains low concentrations of calcium to prevent differentiation) or in RM+ media. All cell lines were cultivated at 37°C and 6% CO_2_. For global demethylation of DNA, N/TERT cells were treated with 5-aza-2′-deoxycytidine (5-Aza) (Sigma–Aldrich, Steinheim, Germany) diluted in dimethyl sulfoxide (DMSO) with different final concentrations of 0 or 10 μM for 72 h with media renewal every 24 h. Differentiation of N/TERT cells grown in KGM-Gold media was accomplished by treatment with 2 mM CaCl_2_ for up to 8 days with 24 h media renewal.

### siRNA Transfection

Silencing of PI(4,5)P_2_ generating kinase gene expression was achieved through transfection of siRNA-SMARTpools (Dharmacon, GE Healthcare, Freiburg, Germany) with the RNAiMAX kit (Thermo Scientific, Waltham, MA, United States) according to the manufacturer’s protocol (siPIP4KIIα: M-006778-01; siPIP4KIIβ: M-006779-03; siPIP5KIα: M-004780-02; siPIP5KIγ: M-004782-00). These siRNAs have previously been successfully used to decrease kinase expression (Bultsma et al., 2010; Chao et al., 2010). The siRNA against Syntenin-2 had the following sequence: siSyn-2: GCAACGGGCTCCTCACCAATT. Cells transfected with a scramble siRNA (Cat. Nr.: 98129_A, Biospring, Frankfurt, Germany) were used as controls.

### Western Blotting

For Western blot analysis, cells were trypsinized, pelleted by centrifugation and lysed on ice in LSDB buffer [50 mM Tris/HCl (pH 8.0), 20% Glycerol, 100 mM KCl, 0,1% NP40, 1 mM DTT, 50 mM NaF, 1 mM orthovanadate, 1 mM PMSF] supplemented with 1× Cocktail Protease Inhibitors (Roche Diagnostics, Mannheim, Germany). The resulting extracts were sonicated, and protein concentration was determined using the Biorad Protein Assay (Biorad, Munich, Germany). Cell extracts were resolved by SDS–PAGE and transferred to a nitrocellulose membrane. After the membrane had been blocked with 5% skimmed milk in TBST [10 mM Tris/HCl (pH 8.0), 150 mM NaCl, 0.05% Tween 20] for 1 h, the blots were probed with antibodies against Syntenin-2 (rabbit polyclonal, Cat. no. 10407-1-AP, diluted 1:1500; Proteintech, Chicago, IL, United States), Loricrin (rabbit polyclonal, Cat. no. ab85679, diluted: 1:1000, Abcam, Cambridge, United Kingdom), PIP5K1α (rabbit polyclonal, Cat. no. PA5-29405, diluted 1:500, Thermo Scientific) and Tubulin (rat monoclonal, Cat. no. YL1/2, diluted 1:10,000; Abcam), which was used as loading control. Immunoreactive proteins were visualized using horseradish peroxidase-coupled IgG secondary antibodies (Dako, Hamburg, Germany) and the BM Chemiluminescence Blotting Substrate Detection System (Roche Diagnostics). The blots were exposed to autoradiographic films and signals were quantified using the ImageJ software.

### Immunofluorescence Staining of Skin Samples

Anonymized EV skin lesions were obtained from routine surgical excisions. The use of the EV tissue for “studies on the mechanisms of HPV oncoprotein action in cutaneous oncogenesis” was approved by the local ethics-committee at the Medical University of Warsaw. HPV typing of these lesions was performed as described in Heuser et al. (2016). Four-micrometer formalin-fixed, paraffin-embedded sections were deparaffinized with xylene. Samples were hydrated through a descending alcohol series (100, 90, and 70%; 5 min each) and endogenous peroxidases were inactivated by incubation in 3% H_2_O_2_ in Methanol for 20 min. Antigen unmasking was performed by boiling the tissue sections in 10 mM citric acid buffer for 3 min in a beaker in a microwave followed by 15 min resting at RT. Further incubation steps were performed in a humid chamber to prevent drying-out. Blocking of unspecific antigen sites was achieved with 50% goat serum (Thermo Scientific) in PBS for 1 h at RT. Incubation with primary antibody against rabbit-anti-Syntenin-2 (ProteinTech) was done in a dilution of 1:250 in 2% goat serum over night at 4°C, followed by three washes with PBS. Sections of EV lesions were stained with rabbit anti-HPV8 E4 antibodies raised against a glutathione S-transferase (GST)-E1^∧^E4 fusion protein (Borgogna et al., 2012). Detection of primary Syntenin-2 antibody was achieved by incubating the sections with a HRP-conjugated goat-anti-rabbit-IgG antibody (Dako, Santa Clara, CA, United States) diluted 1:2000 in 2% goat serum for 1 h at RT, followed by three washes with PBS. The fluorescence staining was performed with the “TSA^TM^-Plus Fluorescein System” (PerkinElmer, Waltham, MA, United States). For E4 detection, sections were incubated with anti-rabbit secondary antibody conjugated with Alexa Fluor 488 (1:500 dilution in 2% FCS; Invitrogen) for 1 h at room temperature. The sections were counterstained with DAPI and embedded with Immumount (Thermo Fisher Scientific, St. Leon-Rot, Germany). Specific signals were visualized on the Leica DMI 6000B fluorescence microscope.

### Quantitative Reverse Transcription-PCR (qRT-PCR)

RNA was isolated from cells using the RNeasy kit, and DNase digestion was performed on-column using RNase-free DNase according to the manufacturer’s instructions (Qiagen, Hilden, Germany) (Lazic et al., 2011; Hufbauer et al., 2015). One microgram of total RNA was reverse transcribed using the Omniscript RT kit (Qiagen, Hilden, Germany) with 10 μM random nonamers (TIB MOLBIOL, Berlin, Germany) and 1 μM oligo(dT_23_) primer (Sigma), as well as 10 units of RNase inhibitor (Thermo Fisher Scientific). Quantitative PCR (qPCR) was performed using the Light-Cycler system (Roche Diagnostics). The total copy number of target genes was normalized to the total copy number of the housekeeping gene encoding hypoxanthine phosphoribosyltransferase 1 (HPRT1). One PCR mixture contained 2 μl of 1:10 diluted cDNA in a total volume of 20 μl, 1.25 units Platinum *Taq* polymerase and the buffer provided in the kit (Invitrogen, Karlsruhe, Germany), 4 mM MgCl_2_, 1.6 μl of a 1:1000 dilution of SYBR^®^ Green (Sigma), 5% DMSO, 0.5 μM each forward and backward primer, 500 ng/μl non-acetylated bovine serum albumin (Thermo Fisher Scientific), and 0.2 mM deoxynucleotide triphosphate. To generate absolute standards, PCR fragments, amplified with primers also used for subsequent qPCR analysis, were cloned into pJET1.2 (Thermo Fisher Scientific). Samples were analyzed in duplicates together with a 10-fold dilution series of standard plasmid. The cycling protocol conditions were 60 s at 95°C, followed by 40 cycles of 1 s at 95°C (20°C/s), 5 s at annealing temperature (melting temperature of primer minus 5°C) (20°C/s), and 15 s at 72°C (20°C/s). The primers used in this study had the following 5′-to-3′ sequences:

Syntenin-2-fw: GTGGACGGGCAGAATGTTAT,
Syntenin-2-bw: ATGGAGATTCTGGCCACG;
HPRT1-fw: CCTAAGATGAGCGCAAGTTGAA,
HPRT1-bw: CCACAGGACTAGAACACCTGCTAA;
PIP4KIIα-fw: ATGGAATTAAGTGCCATGAAAAC,
PIP4KIIα-bw: GCATCATAATGAGTAAGGATGTCAAT;
PIP4KIIβ-fw: TGCATGTGGGAGAGGAGAGT,
PIP4KIIβ-bw: TCAGCTGTGCCAAGAACTCA;
PIP5KIα-fw: CCAACATAAAGAGGCGGAAT,
PIP5KIα-bw: AGGGTTCTGGTTGAGGTTCAT;
PIP5KIγ-fw: AAGGAGGCCGAGTTCCTG,
PIP5KIγ-bw: CGGGTTCTGGTTGAGGTTC;
HPV8-E6-fw: CCGCAACGTTTGAATTTAATG,
HPV8-E6-bw: ATTGAACGTCCTGTAGCTAATTCA.

### Immunocytochemistry

Cells were seeded on coverslips and fixed in 4% paraformaldehyde for 10 min at RT and further permeabilized with 0.1% Triton X-100 for 5 min at RT. After blocking in 10% goat serum (diluted in PBS) for 30 min at RT, cells were incubated with primary antibodies against Syntenin-2 (ProteinTech) diluted 1:250 in 2% goat serum over night at 4°C. On the following day cells were incubated with corresponding fluorescently labeled secondary antibodies, counterstained with DAPI and embedded using Immumount (Thermo Fisher Scientific). Fluorescent signals were visualized on a Leica DMI 6000B fluorescence microscope.

### Statistical Analysis

All qRT-PCR experiments were repeated a minimum of three times in duplicates. The results are expressed as mean ± SEM. Western blots were repeated in *n* = 3 independent experiments. The data presented as immunoblots or images of immunofluorescence analysis are from a representative experiment, which was qualitatively similar in the replicate experiments. Statistical significance was determined with unpaired 2-tailed Student’s *t*-test. The asterisks shown in the figures indicate significant differences of experimental groups (^∗^*p* < 0.05; ^∗∗^*p* < 0.01; ^∗∗∗^*p* < 0.001). Non-significant changes are labeled as “ns.”

## Results

### Papillomavirus Evolution Corresponds with Syntenin Gene Duplication

**FIGURE 1:**
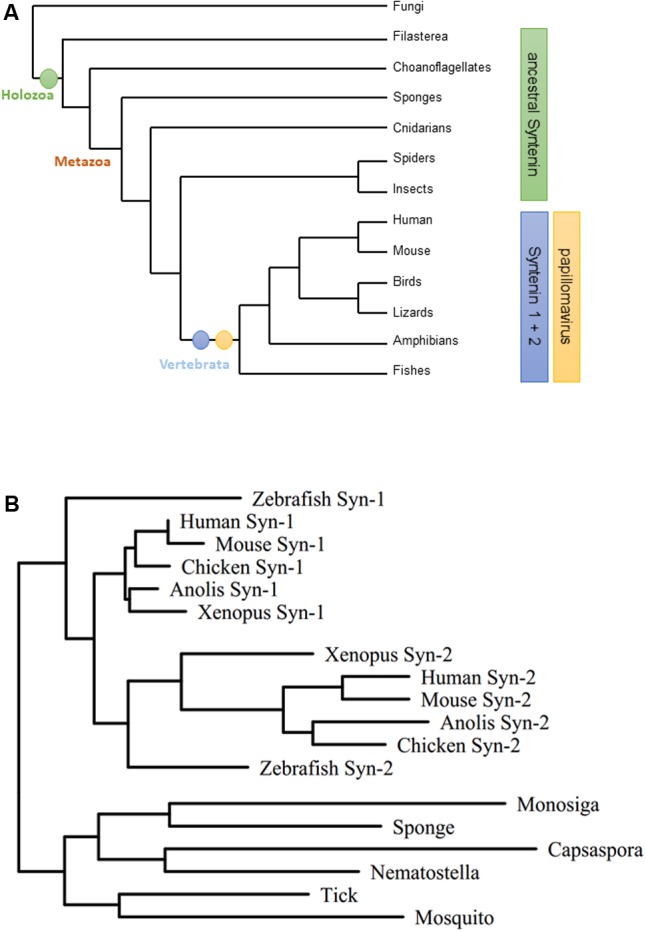
Evolution of the Syntenin gene family. **(A)** Phylogram of major opisthokont lineages [tree simplified after (Torruella et al., 2015)]. Host range of papillomaviruses corresponds to Syntenin-1/Syntenin-2 evolutionary split. **(B)** Maximum likelihood tree for the Syntenin gene family. Representative Syntenin sequences were obtained from UniProt (The UniProt Consortium, 2017), aligned by the L-Ins-I algorithm of the MAFFT package (Katoh et al., 2002), and subjected to tree calculation by the T-Rex server (Boc et al., 2012) using the RAxML method (Stamatakis, 2006) (Syn-1: Syntenin-1; Syn-2: Syntenin-2).

The PDZ protein family of Syntenins is comprised of two family members, Syntenin-1 and Syntenin-2. Retrieving Syntenin sequences from UniProt and constructing phylogenetic trees, we identified that both genes are the result of a gene duplication event that occurred during vertebrate evolution from a single ancestral Syntenin gene. Since the host range of papillomaviruses seems to be restricted to vertebrates (Rector and Van Ranst, 2013; Lopez-Bueno et al., 2016), the proposed timing of Syntenin gene duplication correlates with the evolution of the first papillomaviruses (**Figure 1A**). In addition, Syntenin-1 and Syntenin-2 show extensive structural similarity within the different vertebrate lineages (**Figure 1B**). Thus, Syntenin gene duplication may have contributed to both divergent and convergent evolution of Syntenin-1 and Syntenin-2 during vertebrate evolution.

### Differentiation Dependent Syntenin-2 Expression Is Repressed by betaPV

**FIGURE 2:**
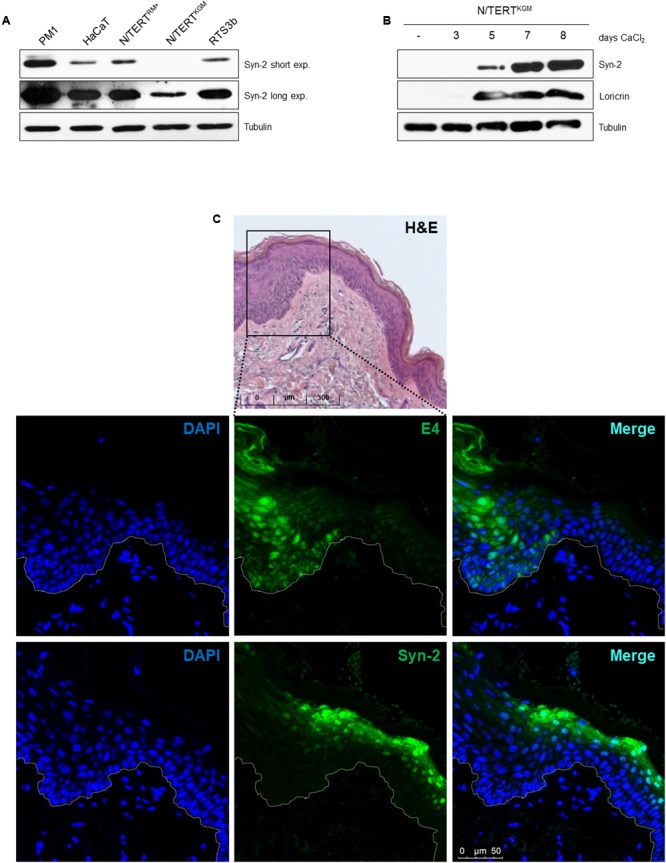
Differentiation dependent expression of Syntenin-2 in skin keratinocytes. **(A)** Representative Western blot showing Syntenin-2 levels in total cell extracts of the keratinocyte cell lines PM1, HaCaT, RTS3b and isogenic N/TERT cells either cultured in RM+ (N/TERT^RM+^) or in KGM-Gold (N/TERT^KGM^). Equal loading was confirmed by immunoblotting for Tubulin. **(B)** Representative Western blot of Syntenin-2 levels in total cell extracts of N/TERT^KGM^ treated with 2 mM CaCl_2_ for up to 8 days. Calcium induced keratinocyte differentiation was confirmed by Western blotting for Loricrin. Equal loading was confirmed by immunoblotting for Tubulin. **(C)** Representative immunofluorescence staining images detecting Syntenin-2 in EV-derived skin lesions. The skin area with productive betaPV infection was identified by anti-E4 staining. Nuclear counterstain was performed using DAPI. Syntenin-2 expression is present in suprabasal keratinocytes of non-lesional/E4-negative EV skin and absent in an E4-positive skin tumor. Top lane: H&E staining of the EV tissue analyzed.

To address the mechanisms through which HPV8-E6 regulates gene expression in a cell line model, Syntenin-2 protein levels were determined in different keratinocyte cell lines. Total cell extracts of the HPV-negative PM1, HaCaT and RTS3b cells were analyzed by Western blotting in addition to N/TERT cells grown either in RM+ (N/TERT^RM+^) or in KGM-Gold (N/TERT^KGM^) media. As shown in **Figure 2A**, PM1, HaCaT and RTS3b cells showed expression of Syntenin-2. However, compared to isogenic N/TERT^RM+^ cells, Syntenin-2 was found to be downregulated in N/TERT^KGM^. These data indicated that Syntenin-2 expression is culture media and differentiation dependent. To further confirm differentiation dependence, N/TERT^KGM^ were exposed to high extracellular calcium concentrations (2 mM) for up to 8 days. Parallel to the induction of the differentiation marker loricrin, confirming the differentiation-inducing cell culture conditions, Syntenin-2 was found to be upregulated in a calcium- and time-dependent manner (**Figure 2B**).

To further evaluate the expression pattern of Syntenin-2 in skin tumors induced by betaPV infection, we stained HPV8 positive skin tumors of EV patients. Staining against the viral E4 protein, which can have both a nuclear and cytoplasmic staining pattern in betaPV-induced tumors was used as surrogate marker for betaPV infection. To visualize E4, a broadly cross-reactive polyclonal antibody raised against the E4 protein of HPV5 and HPV8 was used (Borgogna et al., 2012). As demonstrated in **Figure 2C**, Syntenin-2 is expressed in the cytoplasm and nuclei of suprabasal keratinocytes in E4 negative EV skin and is absent in E4 positive lesional skin. Taken together, these findings implicated that Syntenin-2 expression is dependent on cell differentiation and is downregulated in skin with productive betaPV infection.

### HPV8-E6 Represses Syntenin-2 Expression in Keratinocytes with a Basal Cell Phenotype

**FIGURE 3:**
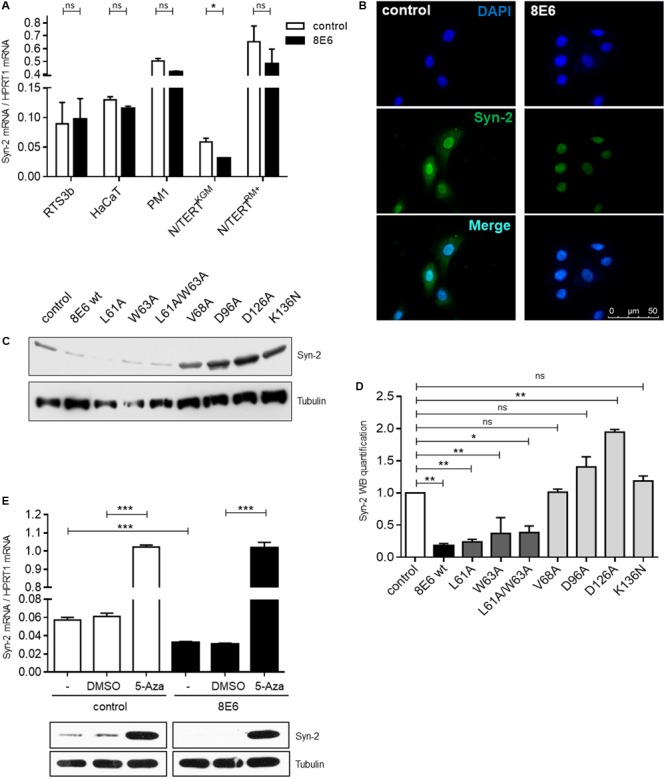
Syntenin-2 expression is repressed by HPV8-E6 in keratinocytes with basal cell characteristics through promoter hypermethylation. **(A)** Quantification of Syntenin-2 mRNA expression by qRT-PCR in empty vector and HPV8-E6 expressing RTS3b, HaCaT, PM1 and isogenic N/TERT keratinocytes cultured either in RM+ or KGM. **(B)** Representative immunocytochemical staining of Syntenin-2 in N/TERT^KGM^-control (Left) and N/TERT^KGM^-8E6 (Right) demonstrating reduction of mainly cytoplasmic Syntenin-2 in HPV8-E6 positive cells (blue: DAPI; green: Syntenin-2). **(C)** Representative Western blot showing Syntenin-2 in total cell extracts of N/TERT^KGM^ expressing either HPV8-E6 wild type or the E6 mutants L61A, W63A, L61A/W63A, V68A, D96A, D126A or K136N. Loading was confirmed by immunoblotting for Tubulin. **(D)** Quantification of Syntenin-2 protein levels from **(C)** normalized to Tubulin protein levels. **(E)** Quantification of Syntenin-2 mRNA expression in N/TERT^KGM^-control and N/TERT^KGM^-8E6 cells after treatment with 10 μM 5-Aza or DMSO (*n* = 3 independent experiments, measured in duplicate, Upper). Data are presented as mean ± SEM (^∗∗∗^*p* < 0.001). Western blot analysis of Syntenin-2 in extracts from cells treated with 5-Aza. Equal loading was confirmed by immunoblotting for Tubulin (Lower).

Given that HPV8-E6 mediated Syntenin-2 repression was previously identified in primary human keratinocytes (Lazic et al., 2012), we next tested whether E6 may also interfere with Syntenin-2 regulation in keratinocyte cell lines. As shown in **Figure 3A**, E6 mediated repression of Syntenin-2 transcription was only found to be significant in N/TERT^KGM^ and was not observed in HPV8-E6 positive N/TERT^RM+^, RTS3b, HaCaT and PM1 cells. Immunocytochemical analysis revealed nuclear and cytoplasmic localization of Syntenin-2 in empty vector transduced N/TERT^KGM^ keratinocytes and a marked reduction of mainly cytoplasmic Syntenin-2 in HPV8-E6 positive cells (**Figure 3B**). To further elucidate the role of conserved amino acids in HPV8-E6 involved in regulation of Syntenin-2 expression, seven mutants of E6 were generated. To test whether repression of Syntenin-2 is a direct consequence of the previously described function of E6 to inhibit Mastermind-like protein 1 (MAML1) dependent differentiation processes (Brimer et al., 2012; Tan et al., 2012; Meyers et al., 2013), the mutants L61A, W63A and L61A/W63A were used. We could show in another study, that the E6 mutants L61A and W63A are still able to bind to MAML1 whereas the double mutant L61A/W63A is deficient in MAML1 binding (own unpublished data). Based on previously published data on HPV5-E6 mutants, the HPV8-E6 mutants V68A, D96A and D126A represent functional mutants, which are still able to inhibit UV-induced apoptosis but are incapable to induce proteasomal degradation of the pro-apoptotic protein Bak (Simmonds and Storey, 2008). The HPV8-E6-K136N mutant is, in contrast to wild type E6, deficient in inhibiting UV-induced DNA damage repair (Hufbauer et al., 2015). In line with wild type E6, the mutants L61A, W63A and the double mutant L61A/W63A (all positions are located within the first zinc-finger) were able to repress Syntenin-2 expression, whereas mutations located near the C-terminus (V68A, D96A, D126A, K136N) failed to inhibit Syntenin-2 expression (**Figures 3C,D**). This data provided evidence that E6 mediated downregulation of Syntenin-2, is a mechanism that is independent of E6 mediated blockade of MAML1 dependent differentiation processes.

### Syntenin-2 Gene Repression in HPV8-E6 Cells Is Released by 5-Aza-2′-deoxycytidine

Hypermethylation of promoter regions can be involved in transcriptional regulation of cellular genes and E6 mediated promoter hypermethylation has previously been described for HPV16-E6 (D’Costa et al., 2012; Schütze et al., 2015; Yin et al., 2017). To test the hypothesis that Syntenin-2 promoter activity may also be methylation-dependent, the effect of 5-Aza-2′-deoxycytidine (5-Aza) mediated inhibition of DNA methylation on Syntenin-2 promoter regulation was measured in the presence and absence of HPV8-E6. E6 expression in cells used for these assays was confirmed by qRT-PCR (**Supplementary Figure S1**). Syntenin-2 mRNA levels were low in DMSO treated cells but significantly increased following treatment with 5-Aza in control cells. This increase in mRNA was also mirrored on the protein level (**Figure 3E**). Syntenin-2 mRNA levels were lower in HPV8-E6 expressing cells compared to empty vector positive and DMSO treated keratinocytes. Syntenin-2 transcription levels were increased about 30-fold following 5-Aza treatment in E6 positive cells, restoring them to the same level that was detected in 5-Aza treated control cells (**Figure 3E**). This complete restoration supports hyper-methylation dependent regulation of Syntenin-2 expression by HPV8-E6.

### Phosphatidylinositol Metabolic Pathway Controls Syntenin-2 Gene Expression

**FIGURE 4:**
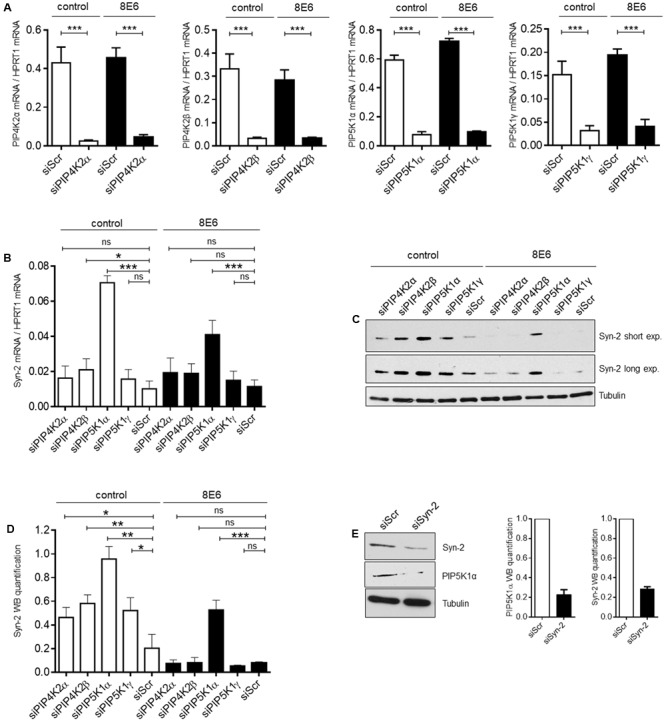
PI(4,5)P_2_ generating kinases are involved in control of Syntenin-2 expression. **(A)** Control (siScr), siPIP4KIIα, siPIP4KIIβ, siPIP5KIα or siPIP5KIγ transfected N/TERT^KGM^-control and N/TERT^KGM^-8E6 cells were harvested 48 h post transfection and mRNA expression of kinase isoforms was determined by qRT-PCR (*n* = 3 independent experiments, measured in duplicate). Data are presented as mean ± SEM (^∗∗∗^*p* < 0.001). **(B)** Quantification of Syntenin-2 mRNA expression by qRT-PCR in N/TERT^KGM^-control and N/TERT^KGM^-8E6, in which the kinase isoforms PIP4KIIα, PIP4KIIβ, PIP5KIα and PIP5KIγ were silenced by siRNA transfection. **(C)** Representative Western blot showing Syntenin-2 expression in total cell extracts from N/TERT^KGM^-control and N/TERT^KGM^-8E6, in which kinase isoforms were silenced by siRNA transfection. Equal loading was confirmed by immunoblotting for Tubulin. **(D)** Quantification of Syntenin-2 protein levels in cells treated with siRNAs for specific kinase isoforms (*n* = 3 independent experiments). Data are presented as mean ± SEM. **(E)** Representative Western blot demonstrating downregulation of PIP5KIα protein levels in cells transfected with specific siRNA against Syntenin-2 (Left). Bars presenting the quantification of PIP5KIα and Syntenin-2 protein levels are shown in the Middle and Right (*n* = 3 independent experiments). Data are presented as mean ± SEM.

It has been shown by Mortier et al. (2005), that nuclear PI(4,5)P_2_ is required for the sub-nuclear enrichment of Syntenin-2. PI(4,5)P_2_ generating kinases are present in the nucleus and have an impact on gene expression by contributing to chromatin unfolding and transcriptional regulation (Delage et al., 2013). In principle, PI(4,5)P_2_ is synthesized by either the phosphorylation of phosphatidylinositol-4-phosphate (PI4P) by the lipid kinase phosphatidylinositol-4-phosphate-5-kinase type I (PIP5KI) or by the phosphorylation of phosphatidylinositol-5-phosphate (PI5P) through phosphatidylinositol-5-phosphate-4-kinase type II (PIP4KII) (van den Bout and Divecha, 2009). Each of these kinases comprises three isoforms, given the designations α, β and γ. To evaluate the functional correlation between the PI(4,5)P_2_ pathway and Syntenin-2 gene expression, we next analyzed the impact of PI(4,5)P_2_ generating kinases on Syntenin-2 expression through silencing of the single kinase isoforms. The kinase isoform PIP4KIIγ is not a nuclear protein and was therefore not studied (Itoh et al., 1998; Clarke et al., 2008) in addition to PIP5KIβ, which is not expressed in N/TERT^KGM^ (data not shown). A strong knockdown of the kinase isoforms could be achieved for all four tested enzymes using kinase specific siRNA pools in E6 negative and positive cells (**Figure 4A**). The expression of HPV8-E6 in these cells was confirmed by qRT-PCR and was not significantly changed in all siRNA treated cells (**Supplementary Figure S2**). Knockdown of PIP4KIIβ and particularly PIP5KIα led to a significant upregulation of Syntenin-2 mRNA expression in wild type keratinocytes. Interestingly, HPV8-E6 was able to counteract siPIP4KIIβ and siPIP5KIα mediated re-activation of Syntenin-2 transcription (**Figure 4B**). On protein level, kinase silencing led to the induction of Syntenin-2 expression in siPIP4KIIα, siPIP4KIIβ, siPIP5KIα and siPIP5KIγ transfected cells with siPIP5KIα showing the strongest effect in empty vector positive N/TERT^KGM^ cells. HPV8-E6 blocked Syntenin-2 protein re-expression in siPIP4KIIα, siPIP4KIIβ and siPIP5KIγ treated cells and counteracted siPIP5KIα-mediated re-expression of Syntenin-2 by 50% (**Figures 4C,D**). To further determine whether, on the other hand, Syntenin-2 is a regulator of PIP5K1α expression, we also measured protein levels in keratinocytes treated with Syntenin-2 specific siRNA. Unexpectedly, knockdown of Syntenin-2 led to a strong downregulation of PIP5K1α protein expression (**Figure 4E**).

## Discussion

We recently identified Syntenin-2 as the first PDZ protein to be targeted by HPV8-E6 on transcriptional level in primary keratinocytes cultured in low calcium media (Lazic et al., 2012). In this study we elucidated the mechanisms controlling Syntenin-2 gene expression. We now could show that Syntenin-2 is expressed in different keratinocyte cell lines cultured in RM+ media. It was, however, significantly downregulated in N/TERT cells grown in KGM media, under cell culture conditions in which keratinocytes do not differentiate and phenotypically resemble basal epidermal cells. Using N/TERT^KGM^ keratinocytes as a cell line model, we demonstrate that HPV8-E6 is capable of suppressing Syntenin-2 expression only in these keratinocytes. We previously demonstrated, that Syntenin-2 is absent in three-dimensional organotypic skin cultures of HPV8-E6 positive keratinocytes, which show loss of normal stratification and the absence of stratum corneum formation. In addition, downregulation of Syntenin-2 through shRNA expression inhibited differentiation of normal keratinocytes in skin cultures (Lazic et al., 2012). The interference of E6 with normal keratinocyte differentiation represents an oncogenic mechanism that has recently been linked to the ability of HPV8-E6 to bind to MAML1 and thus blocks NOTCH-dependent differentiation regulation (Brimer et al., 2012; Tan et al., 2012; Meyers et al., 2013). HPV8-E6 is also known to prevent pro-Caspase-14 cleavage, which is involved in the regulation of late terminal differentiation of keratinocytes (Kazem et al., 2011). We now show, that Syntenin-2 expression is absent in suprabasal cell layers of HPV8 positive EV skin tumors. The fact that the L61A/W63A mutant, which is not able to bind to MAML1 still represses Syntenin-2 indicated that Syntenin-2 downregulation is not linked to E6-mediated inhibition of MAML1/NOTCH-dependent differentiation. This led to the conclusion that reduction of Syntenin-2 by E6 may contribute to betaPV-mediated alteration of keratinocyte differentiation independent of MAML1 inhibition.

Another key observation is, that the Syntenin-2 promoter is regulated by DNA methylation. The observation, that Syntenin-2 mRNA and protein levels could completely be restored by 5-Aza treatment in HPV8-E6 expressing cells, led us to the conclusion, that E6 mediated suppression of Syntenin-2 is mediated through promoter hypermethylation. The exact mechanism how HPV8-E6 induces hypermethylation of cellular gene promoters and how the C-terminal part of E6 contributes to this needs still to be investigated.

**FIGURE 5:**
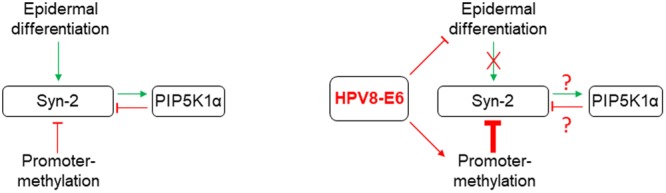
Graphical summary of the regulatory pathways identified in this study controlling Syntenin-2 expression in normal keratinocytes (Left). Known HPV8-E6 mediated interference with these mechanisms is shown on the Right.

Syntenin-2 is known to bind nuclear PI(4,5)P_2_ with high affinity (Mortier et al., 2005). Phosphatidylinositides within the nucleus appear to influence many steps of transcriptional regulation, including reading and writing of the histone code, polymerase-dependent transcription, splicing and polyadenylation, and finally mRNA export (Lewis et al., 2011; Shah et al., 2013). In addition to our observation that differentiation and methylation pathways are involved in Syntenin-2 regulation, we also provide evidence, that Syntenin-2 is a target of the PI(4,5)P_2_ metabolic pathway (**Figure 5**). Particularly, the downregulation of PIP5KIα resulted in re-expression of Syntenin-2 and this specific Syntenin-2 upregulation could only partially be counteracted by E6. Whether shortage of PI(4,5)P_2_, accumulation of PI4P or PIP5KIα functions during keratinocyte differentiation (Xie et al., 2009; Shrestha et al., 2016) contribute to transcriptional repression of Syntenin-2 remains unclear. In addition, the observation that Syntenin-2 silencing using siRNAs resulted in inhibition of PIP5KIα protein expression points to a negative feedback loop between these two factors (**Figure 5**).

Known interaction partners of Syntenin-1 do not interact with Syntenin-2, which may imply that they are functionally not redundant (Koroll et al., 2001). Both Syntenins have evolved by gene duplication from an ancestral Syntenin gene during vertebrate evolution. Gene duplication has been linked to many aspects of genome evolution and is a potent way to create new biological functions (Wagner et al., 2007), which may also account for Syntenin proteins. Syntenin-1 is predominantly located in cell – cell adherens junctions and cytoplasm and is involved in diverse functions including human tumorigenesis (Beekman and Coffer, 2008; Friand et al., 2015; Philley et al., 2016). Interestingly, Syntenin-1 has been linked with the endocytic pathway controlling HPV entry into the host cell, controlling the delivery of internalized viral particles to endosomes and viral capsid disassembly in a CD63-Syntenin-1-ALIX dependent manner (Grassel et al., 2016). Although human Syntenin-2 is highly related to Syntenin-1 in its overall domain organization and sequence, both proteins show higher similarities with the homolog protein of other vertebrate species than to the variants expressed in an individual organism. It is tempting to speculate that during evolution Syntenin-1 may have provided the platform for virus-host specification of an ancestral papillomavirus as a key event in the evolution determinant of papillomaviruses (Gottschling et al., 2011; Van Doorslaer, 2013). However, at the same time, papillomaviruses may need to downregulate Syntenin-2 through E6 to achieve tissue dedifferentiation to enable efficient viral propagation.

## Conclusion

We have advanced the understanding of Syntenin-2 gene regulation in normal cells and how HPV8-E6 can interfere with these mechanisms. Elucidation of the Syntenin-2 regulated pathways will provide further knowledge on its role in regulating keratinocyte differentiation.

## Author Contributions

Conceived and designed the experiments: BM and BA. Performed the experiments: BM, DM-L, KH, and MH. Analyzed the data: BM, DM-L, MH, and BA. Approved the final version of the manuscript: BM, DM-L, KH, SM, JD, MH, and BA. Contributed reagents/materials: SM and JD. Wrote the paper: BA.

## Conflict of Interest Statement

The authors declare that the research was conducted in the absence of any commercial or financial relationships that could be construed as a potential conflict of interest.

## Supplementary Material

FIGURE S1 Measurement of HPV8-E6 mRNA levels by qRT-PCR in N/TERT^KGM^-control and N/TERT^KGM^-8E6 cells treated with DMSO or 5-Aza. Total E6 mRNA was normalized to HPRT1 mRNA levels.

FIGURE S2 Measurement of HPV8-E6 mRNA levels by qRT-PCR in N/TERT^KGM^-control and N/TERT^KGM^-8E6 cells transfected with a Scramble siRNA (siScr) or with siRNAs directed against PIP4KIIα, PIP4KIIβ, PIP5KIα or PIP5KIγ. Total E6 mRNA was normalized to HPRT1 mRNA levels.
