# Supplementary figures and images for: HPV8-E6 Interferes with Syntenin-2 Expression through Deregulation of Differentiation, Methylation and Phosphatidylinositide-Kinase Dependent Mechanisms
Source: Front Microbiol. 2017 Sep 8;8:1724. doi: 10.3389/fmicb.2017.01724 (PMC5609557; doi:10.3389/fmicb.2017.01724)

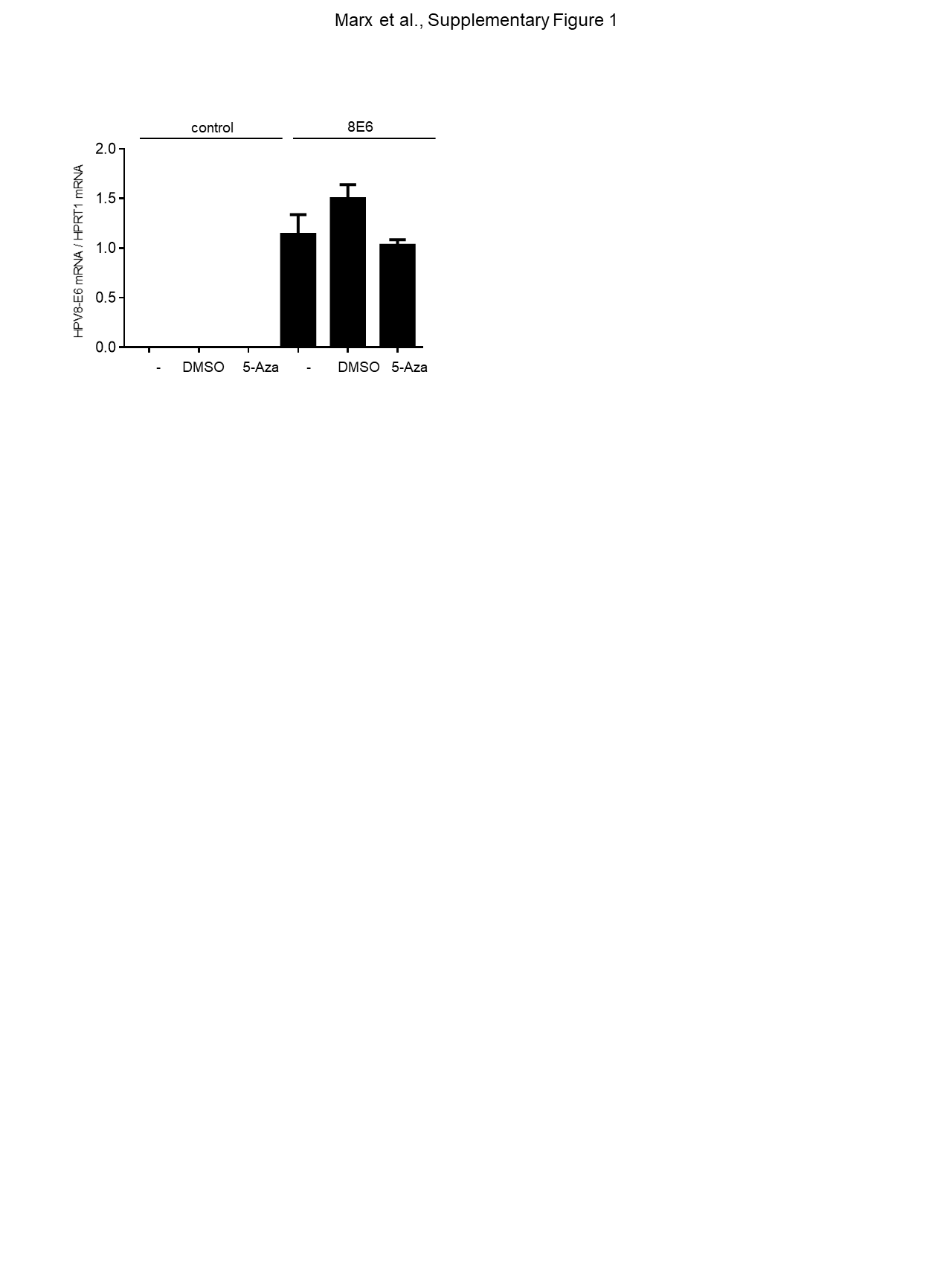

Supplement: FIGURE S1 — Measurement of HPV8-E6 mRNA levels by qRT-PCR in N/TERTKGM-control and N/TERTKGM-8E6 cells treated with DMSO or 5-Aza. Total E6 mRNA was normalized to HPRT1 mRNA levels. [file Image_1.TIF]

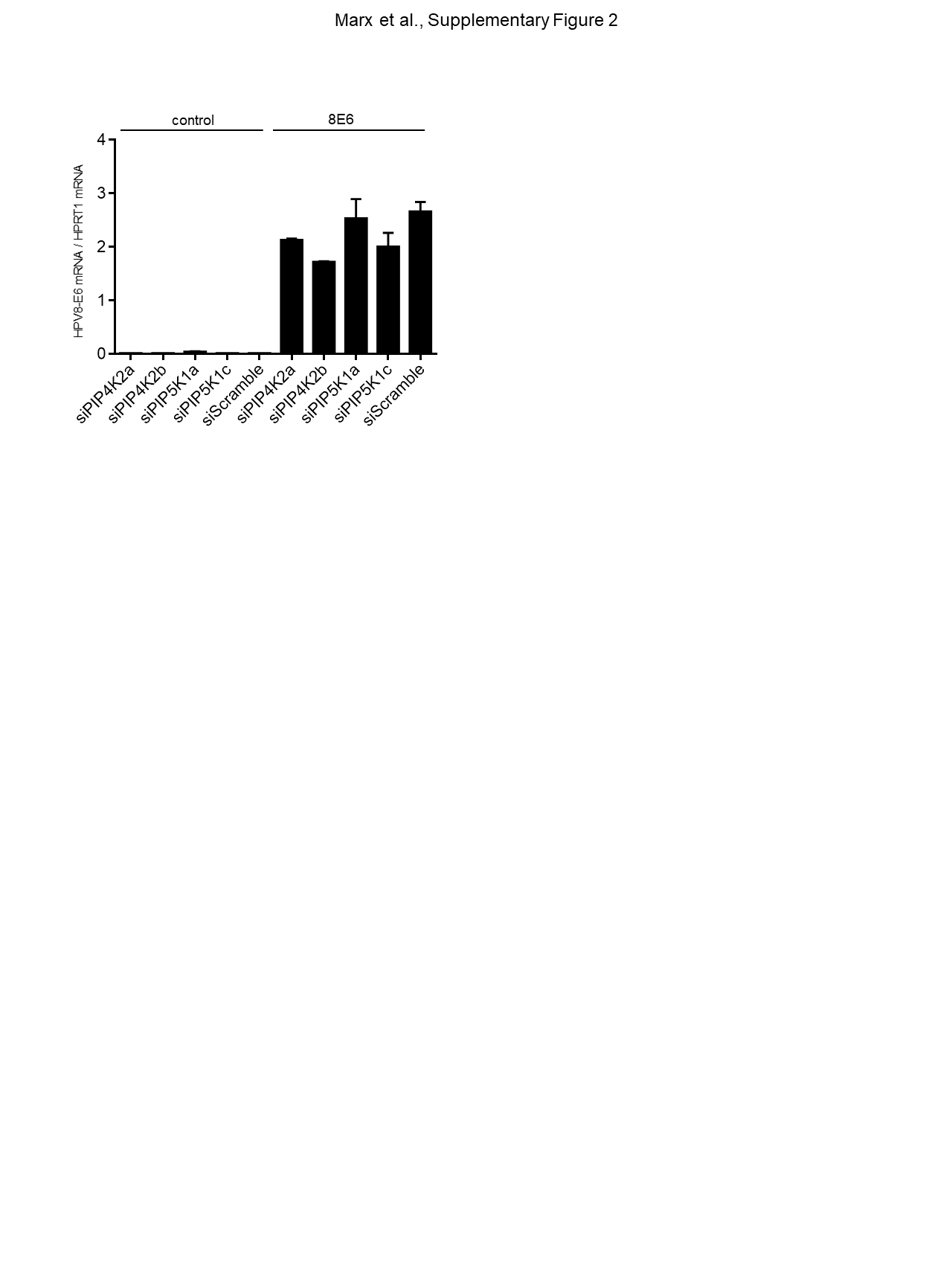

Supplement: FIGURE S2 — Measurement of HPV8-E6 mRNA levels by qRT-PCR in N/TERTKGM-control and N/TERTKGM-8E6 cells transfected with a Scramble siRNA (siScr) or with siRNAs directed against PIP4KIIα, PIP4KIIβ, PIP5KIα or PIP5KIγ. Total E6 mRNA was normalized to HPRT1 mRNA levels. [file Image_2.TIF]
